# Cellulose Hydrogels Derived from Pineapple Bagasse for Potential Dental Applications: Chlorhexidine-Loaded Hydrogels with Antibacterial and Cytocompatible Properties

**DOI:** 10.3390/gels11110891

**Published:** 2025-11-05

**Authors:** Itzel Nevarez-Rico, Guillermo Ignacio Guangorena-Zarzosa, Takaomi Kobayashi, Salvador David Nava-Martínez, Rosa Alicia Saucedo-Acuña, Juan Carlos Cuevas-González, Judith Ríos-Arana, León Francisco Espinosa-Cristobal, María Verónica Cuevas-González, Erasto Armando Zaragoza-Contreras, Karla Lizette Tovar-Carrillo

**Affiliations:** 1 Instituto de Ciencias Biomédicas, Universidad Autónoma de Cd. Juárez, Av. Benjamín Franklin # 4960, Zona Pronaf, Ciudad Juárez C.P. 32315, Chihuahua, Mexico; itzy_nr@hotmail.com (I.N.-R.); Salvador.Nava@uacj.mx (S.D.N.-M.); rosauced@uacj.mx (R.A.S.-A.); cuevas_gonzalez@hotmail.com (J.C.C.-G.); jrios@uacj.mx (J.R.-A.); leon.espinosa@uacj.mx (L.F.E.-C.); maria.cuevas@uacj.mx (M.V.C.-G.); 2 Department of Science of Technology Innovation, Nagaoka University of Technology, 1603-1, Kamitomioka, Nagaoka 940-2188, Niigata, Japan; guillermo.guangorenaz@gmail.com (G.I.G.-Z.); takaomi@vos.nagaokaut.ac.jp (T.K.); 3 Centro de Investigación en Materiales Avanzados, S.C. Miguel de Cervantes No. 120, Complejo Industrial Chihuahua, Chihuahua C.P. 31136, Chihuahua, Mexico

**Keywords:** antibacterial, cellulose hydrogel, cytocompatibility, dentistry, pineapple fiber

## Abstract

Pineapple fibers were used as a sustainable raw material to isolate native cellulose from alkaline–acid treatment. The cellulose fibers were regenerated into transparent and flexible cellulose hydrogels using the lithium chloride/N,N-dimethylacetamide (LiCl/DMAc) solvent system, followed by a phase-inversion process of the cellulose solution under ethanol vapor. Chlorhexidine was incorporated into the hydrogels to provide antibacterial properties. The concentration of chlorhexidine ranged from 0.1 to 0.8 wt%. The prepared hydrogels showed better early onset cytocompatibility than the cell culture dish used as a control. For the evaluation of antibacterial properties, strains of *Streptococcus mutans*, *Streptococcus sanguis*, and *Streptococcus anginosus* were used. The results indicated antibacterial activity at all chlorhexidine concentrations tested, with the area of bacterial inhibition increasing with increasing bactericidal content in the hydrogel films. Adding bactericide into cellulose films did not compromise their early onset cytocompatibility in the first 72 h. The study suggests that adding chlorhexidine provides the hydrogel films with antibacterial properties, potentially expanding their applications in dentistry.

## 1. Introduction

Hydrogels are three-dimensional polymeric networks with a strong affinity for water, allowing them to absorb and retain large amounts of fluid without losing their structural integrity. Because of their hydration capacity, flexibility, and tunable mechanical properties, hydrogels closely mimic the natural extracellular matrix (ECM), providing a favorable environment for cellular adhesion, proliferation, and differentiation [1,2,3]. These materials can be synthesized from natural polymers such as cellulose, chitosan, alginate, and hyaluronic acid, or from synthetic macromolecules including polyacrylamide, polyethylene glycol, and polyvinyl alcohol [4,5,6]. Hydrogels have been widely studied for their potential in wound care, tissue scaffolding, and controlled drug or gene delivery systems. Furthermore, the integration of nanotechnology and advanced fabrication techniques, such as 3D bioprinting and nanoparticle embedding, has significantly improved their structural performance, bioactivity, and responsiveness to physiological stimuli [7,8,9,10].

Among natural hydrogels, cellulose-based systems have gained considerable interest due to their biodegradability, biocompatibility, and structural similarity to the ECM. Their inherent hydrophilicity supports cell attachment and facilitates the controlled release of therapeutic agents [11,12,13,14].

From a sustainability standpoint, agricultural and agroindustrial residues provide an abundant and renewable source of cellulose for the fabrication of functional hydrogels. By-products such as rice husks, sugarcane bagasse, corn husks, agave fibers, and pineapple residues are rich in cellulose and can be valorized to produce high-performance biomaterials, contributing to circular economy models [15,16]. Among these, pineapple waste, including peels, leaves, and cores, represents a particularly promising raw material because of its availability and high cellulose yield [17,18]. Cellulose isolated from pineapple residues can be transformed into hydrogels with controllable porosity, swelling behavior, and mechanical stability, providing environments conducive to cell attachment and proliferation [19]. Functionalization of these matrices with antimicrobial compounds, nanoparticles, or growth factors further broadens their use in biomedical fields such as wound healing, tissue regeneration, and controlled drug release [20,21,22,23].

In dental applications, the need for materials that exhibit strong biocompatibility and support for cell attachment is essential. However, the oral cavity presents a particularly challenging environment due to constant microbial exposure. Therefore, hydrogels with inherent antibacterial activity are desirable to reduce bacterial colonization on restorative or coating materials [24,25,26,27,28,29,30,31,32]. Chlorhexidine, a broad-spectrum antiseptic effective against Gram-positive and Gram-negative bacteria, is among the most widely used antimicrobial agents in dentistry. It functions by interacting with microbial cell membranes, causing disruption and leakage of cellular components [30,31,32]. Due to these properties, it is extensively used as a disinfectant in wound care and oral health treatments, including plaque control and the management of fungal infections [30].

Incorporating chlorhexidine into cellulose hydrogels merges the biocompatibility and structural properties of the polymeric matrix with the antimicrobial efficacy of the drug [33]. Such systems enable localized and sustained release, maintaining prolonged antibacterial activity while reducing systemic exposure. Previous reports have demonstrated that chlorhexidine-loaded cellulose hydrogels effectively prevent biofilm formation and microbial proliferation on wounds and medical devices, showing promise in both wound management and oral applications where controlled antimicrobial delivery is crucial [34].

In this work, we developed cellulose hydrogel films derived from pineapple bagasse, an abundant byproduct of the food industry. Valorizing this residue not only addresses waste management and environmental concerns but also supports the creation of sustainable biomaterials for biomedical use [35,36,37,38,39,40,41]. The hydrogels produced from bagasse-derived cellulose exhibited high water retention, interconnected nanofibrillar porosity, and excellent compatibility with biological systems, features that are suitable for encapsulating hydrophilic antimicrobial agents, such as chlorhexidine [42]. In this first study, we are evaluating the antibacterial performance, early onset cytocompatibility and biocompatibility of chlorhexidine-loaded cellulose hydrogels for potential dental applications [29,30,31,32]. To our knowledge, this is the first report describing the synthesis and evaluation of cellulose hydrogels from pineapple bagasse as a platform for the delivery of an antibacterial agent. The proposed material underscores the potential of agricultural waste valorization while offering a promising, eco-friendly alternative for biomedical and dental therapies.

## 2. Results and Discussion

### 2.1. Preparation of Pineapple Cellulose with Chlorhexidine

To prepare hydrogels from pineapple waste fibers, the fibers were washed to eliminate sugar traces, then the fibers were dissolved in DMAC solution with LiCl, and then solvent exchange was performed between water and ethanol. Chlorhexidine content was varied from 0.1 to 0.8 wt%. Table 1 shows the shear viscosity of cellulose solutions, varying the chlorhexidine content, as measured at 6 and 60 rpm. As observed, when the amount of chlohexidine increased, shear viscosity decreased. This was attributed to the reduction in the interaction of the LiCl/DMAc with the cellulose fibers due to the presence of chlorhexidine in the solution [43].

Additionally, Table 1 lists the properties of wet hydrogel films. For water adsorption, equilibrium was reached after 36 h. As noted, the amount of water adsorbed decreased from 34% to 22% with an increase in chlorhexidine in the hydrogel. Apparently, the bactericide reduces the cellulose’s hydrophilicity.

Regarding the contact angle, it was observed that at higher concentrations of chlorhexidine, water molecules were less able of penetrating the hydrogel film, resulting in a larger contact angle. Moreover, all the obtained hydrogel films are very soft and flexible, although no chemical crosslinking was used.

Table 1 also summarizes the tensile strength and elongation properties of the hydrogel films. A noticeable decrease in tensile strength, from 0.65 N/mm^2^ to 0.54 N/mm^2^, and in elongation, from 36 mm to 20 mm, was observed as the chlorhexidine content increased. This reduction can be attributed to the incorporation of chlorhexidine, which likely disrupts the intermolecular interactions between cellulose fibers within the film matrix. Consequently, this interference may explain the reduced stiffness observed in samples containing higher chlorhexidine concentrations. Interestingly, the film with the lowest amount of chlorhexidine exhibited the highest tensile strength, possibly due to stronger interactions between cellulose fibers and the macrocations formed in the DMAc/LiCl solvent system. It has been suggested that, in this system, macrocations of the form [Li(DMAc)_x_]^+^ [44] are generated, which interact with cellulose chains and act as physical crosslinkers. The formation of these aggregates, resulting from macrocation-cellulose interactions, has been previously confirmed [43].

A recent comparative study of regenerated cellulose hydrogels prepared via various solvent systems (including LiCl/DMAc) reported tensile strengths of ~0.523 N/mm^2^ for films with ~2% solid content, and compressive elastic moduli on the order of ~0.9 N/mm^2^ [45]. Similarly, cellulose/Fe-reinforced hydrogels exhibited tensile strengths around 0.8–1.3 N/mm^2^ for typical formulations [45]. Also, a cellulose hydrogel prepared via the LiCl/DMAc solvent system was treated with UV radiation. The tensile strength was found to vary between 0.3 and 0.8 N/mm^2^, depending on the irradiation time [46]. As observed, our hydrogel films exhibit mechanical properties comparable to those reported in the literature. This suggests that their structural integrity is suitable for dental and soft tissue applications, which typically require mechanical robustness but not the load-bearing capacity levels of hard tissue implants.

It should be noted that cellulose-based hydrogels exhibit tunable swelling and mechanical behaviour that depend strongly on polymer concentration, fibrillar structure and crosslinking density. These factors govern water uptake and elastic modulus and can be tailored for soft-tissue applications [47]. Similar cellulose films loaded with chlorhexidine show modified mechanical and swelling responses after their incorporation, while retaining their antibacterial function [48]. Therefore, it can be assumed that the mechanical behavior of our films is compatible with applications that require flexibility and moisture retention. However, it is important to emphasize that suitability for dental indications should be supported by mechanical characterization (including rheology and AFM) and in vitro testing under oral-like conditions.

**Figure 1:**
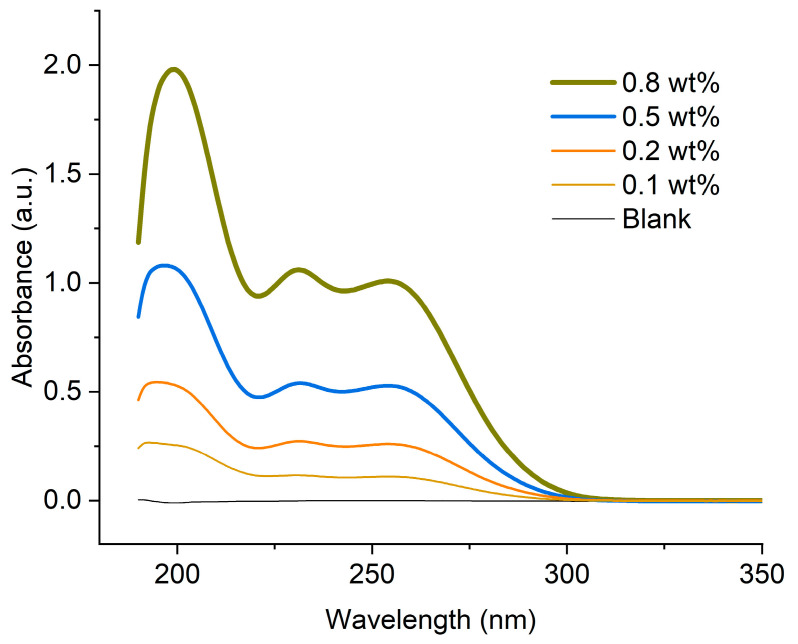
Uv–Vis spectra of cellulose hydrogel films having 1.2 mm thickness. The hydrogel films containing different chlorhexidine from 0.1 to 0.8 wt%.

Figure 1 shows the UV–Vis spectra of hydrogel films containing chlorhexidine (0.0, 0.1, 0.2, 0.5, and 0.8 mg). The absorption spectra of the cellulose hydrogels showed that the chlorhexidine-loaded hydrogel films exhibit absorption bands at 200, 230, and 255 nm [49]. Chlorhexidine is a symmetrical molecule with two p-chloro-phenyl substituted biguanide groups. The intensity of the peaks increased with the increment of chlorhexidine. Therefore, in this study, the absorbance bands in the UV–Vis spectra were used to detect the presence of chlorhexidine in the hydrogel films. It was observed that stronger bands appeared in samples with higher chlorhexidine content. These results were consistent with the various chlorhexidine concentrations reported in the literature [43].

**Figure 2:**
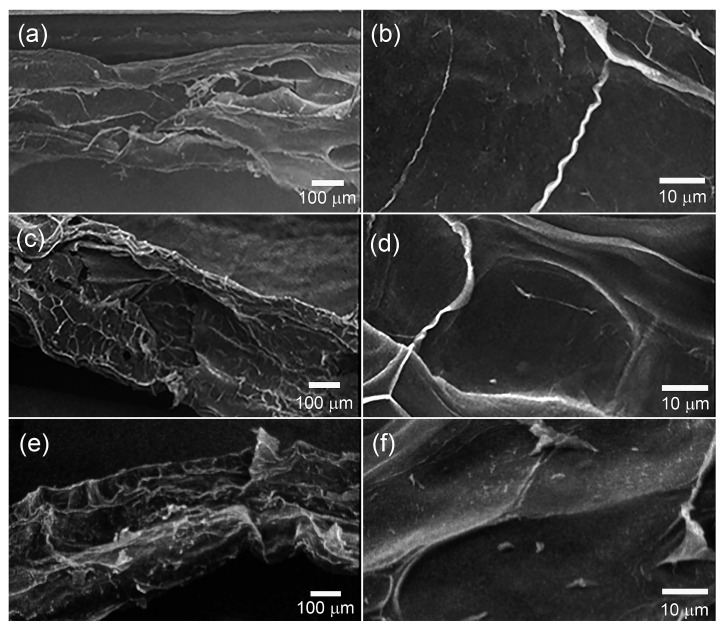
Morphology of the cellulose hydrogels observed by SEM. Images of a pure cellulose hydrogel (**a**,**b**) and cellulose hydrogels containing 0.1 wt% (**c**,**d**), and 0.8 wt% (**e**,**f**).

Figure 2 presents SEM images of hydrogel films without chlorhexidine (Figure 2a,b) and containing 0.10 wt% (Figure 2c,d) and 0.80 wt% (Figure 2e,f) chlorhexidine. Figure 2 shows SEM images of the cellulose hydrogel film, as well as samples prepared with the minimium and maximum chlorhexidine concentrations used in this study. The images revel cross-sections of a homogeneous material and mostly uniform surface. The surface homogeneity of the hydrogel is an important factor for protein adsorption and cell adhesion, which are both crucial for the potential use of the material as a scaffold in tissue regeneration applications.

### 2.2. Antibacterial Activity

In this research, the use of chlorhexidine was considered due to its relevance in dental applications. This compound is widely utilized in various dental treatments, often requiring it to be incorporated or fixed within a material for effective use. Thus, incorporating chlorhexidine into cellulose hydrogel was proposed, as this system can serve as an alternative in multiple dental procedures [30,32]. Cellulose hydrogels containing chlorhexidine could compete with synthetic scaffolds by providing a structure made from agro-industrial waste materials, making it an economical and sustainable alternative [10]. Consequently, this hydrogel may serve as a foundation for developing various materials for dental applications, including drug-releasing membranes, biosensors, or gingival regeneration.

**Figure 3:**
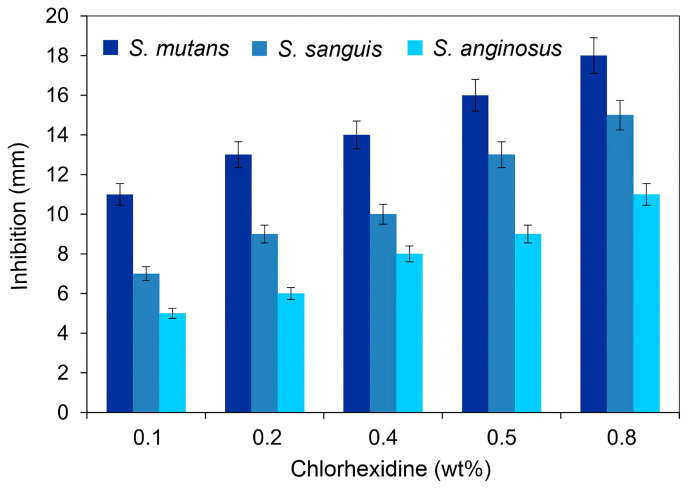
Antibacterial activity of cellulose hydrogels, varying the chlorhexidine concentration. Inhibition halo at 24 h of culture at 37 °C. Data presented in millimeters, mean ± standard deviation, error bars 95% *p* < 0.05, *n* = 6.

Figure 3 illustrates the comparative antibacterial activities of cellulose hydrogels varying the concentration of chlorhexidine using an inhibition zone chart of the tested antimicrobial samples (0.1, 0.2, 0.4, 0.5, and 0.8 wt% chlorhexidine). The results from the disk diffusion method against three types of microorganisms indicated that an increase in the inhibition zone occurred as the chlorhexidine concentration increased. Diffusion test showed inhibition zones for *S. mutans*, *S. sanguis*, and *S. anginosus* [50]. These microorganisms were chosen because they are commonly found in the human oral cavity, contributing significantly to tooth decay and oral disease. Moreover, the presence of chlorhexidine inhibited bacterial growth in a zone of 5 to 18 mm, showing that the most sensitive bacterium to chlorhexidine was *S. mutans*, followed by *S. sanguis* and *S. anginosus*, respectively. It was also found that hydrogel films containing a higher content of chlohexidine had the most effective antimicrobial activity in the first hours of exposure. In this case, the chlorhexidine increment showed higher inhibition zones compared to the rest of the chlorhexidine concentrations. Other works, using chlorhexidine, reported antibacterial activity after 48 and 72 h for 12 weeks as the concentration of the bactericide increased [30,32].

This work shows that the growth inhibition effect in *S. mutans*, *S. sanguis*, and *S. anginosus* depends on the concentration of chlorhexidine in the hydrogel film. Furthermore, the inhibition zones were higher for *S. mutans* with 11 to 18 mm, *S. sanguis* with 7 to 15 mm, and *S. anginosus* with 5 to 11 mm, respectively, depending on chlorhexidine content. These findings exhibit greater inhibition zones, from 5 to 18 mm, in the first hours of exposure of the films with microorganisms, and an increase in inhibition as the chlorhexidine content increases. This tendency was observed in all the samples prepared, varying the chlorhexidine content.

*S. sanguis* and *S. mutans* act in the first stage formation of dental plaque, causing significant pathogenicity compared to the rest of the periodontal bacteria present. It has been reported that highly specific interspecies interactions may have an essential role in the balance of microbial communities with multiple species in the oral cavity. In addition, in vitro evaluations of *S. sanguis* interacting with *S. mutans* have demonstrated a regulated coexistence of their microbiological activity. Thus, having a material with antibacterial activity against *S. sanguis* and *S. mutans* helps reduce the main microorganisms implicated in the formation of dental plaque.

**Figure 4:**
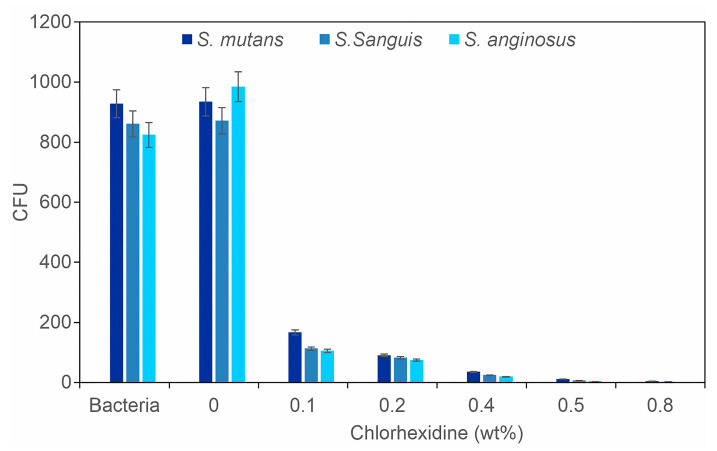
Effect of chlorhexidine concentration on colony formation (CFU) of *S. mutans*, *S. sanguis*, and *S. anginosus* at 24 h of culture at 37 °C, *p* < 0.05, *n* = 6.

In addition to the inhibition halo method, we also conducted a colony-forming unit (CFU) count (Figure 4). The trend in antimicrobial activity depicted in Figure 3 aligns with the CFU measurements. A significant reduction in colony formation was observed with increasing chlorhexidine concentration. This finding is consistent with previous literature [30], which supports the antimicrobial efficacy of mouth rinses containing chlorhexidine based on in vivo studies that utilized samples collected directly from patients and performed colony counts.

### 2.3. Cytotoxicity and Biocompatibility Assays

#### 2.3.1. Protein Adsorption

**Figure 5:**
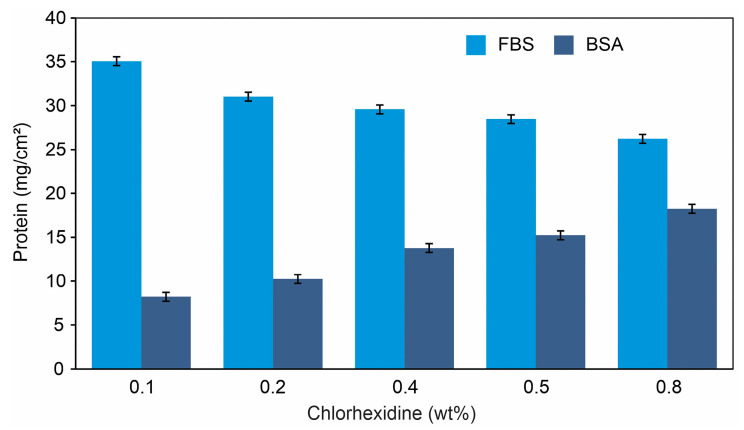
Protein adsorption on hydrogel films (data are presented in mean ± standard deviation), indicating statistical differences between the concentration groups of chlorhexidine, *p* < 0.05, *n* = 6.

For the biocompatibility assessment, cellulose hydrogels with and without chlorhexidine were evaluated through serum protein adsorption tests using bovine serum albumin (BSA) and fetal bovine serum (FBS). Figure 5 presents the protein adsorption results for cellulose hydrogel films containing chlorhexidine. When an artificial scaffold is exposed to cells suspended in a culture medium supplemented with FBS, serum proteins rapidly adsorb onto its surface before subsequent cell adhesion. The results revealed significant differences in protein adsorption as the chlorhexidine content increased (*p* < 0.05). A relationship was found between chlorhexidine concentration and the adsorption of both FBS and BSA. Concerning FBS, it was observed that as the chlorhexidine content in the hydrogel increased, the amount of adsorbed FBS decreased (Student’s *t*-test, *p* < 0.05, *n* = 6). Conversely, an opposite trend was observed for BSA, with a higher amount of BSA adsorption registered as the chlorhexidine amount increased. It is well established that BSA tends to adsorb more strongly onto hydrophobic surfaces, whereas FBS shows a preference for hydrophilic ones [51,52]. In this study, the decrease in FBS adsorption with increasing chlorhexidine concentration apparently correlates with decreased cell adhesion. Thus, variations in BSA and FBS adsorption may reflect changes in the hydrogel’s surface characteristics depending on the chlorhexidine content. Previous reports indicate that early BSA adsorption can hinder cell attachment, whereas exposure to FBS-containing media allows rapid adsorption of serum proteins, facilitating subsequent cell adhesion and counteracting the inhibitory effect of BSA [48]. Based on these findings, cellulose hydrogel films with higher chlorhexidine content would be expected to promote lower levels of cell adhesion due to altered protein–surface interactions.

#### 2.3.2. Cell Adherent Number

To further explore the potential of cellulose hydrogel films for possible dental applications, fibroblast adhesion on their surface was evaluated, as cellular attachment is a key indicator of early onset cytocompatibility and biocompatibility and tissue integration. Phase-contrast optical microscopy was employed to observe fibroblast behavior on the hydrogel surfaces and to determine how chlorhexidine concentration influenced cell density.

**Figure 6:**
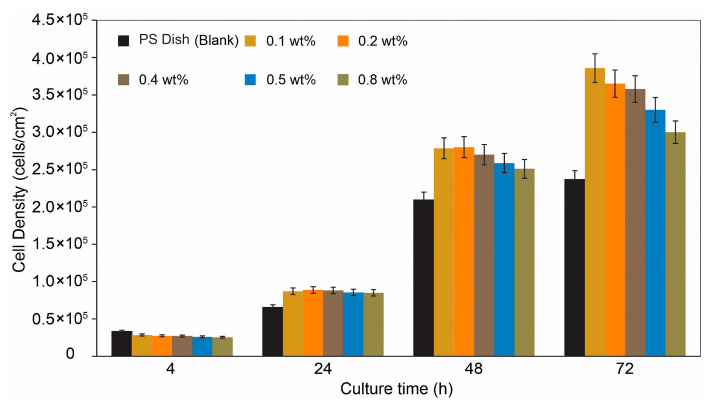
Cell culture experiments using hydrogel films with various concentrations of chlorhexidine. Values indicate statistical differences between the concentration groups of chlorhexidine, *p* < 0.05, *n* = 6. After 72 h of cell culture time, higher cell adherent number was observed on samples with lower chlorhexidine content.

Figure 6 shows the results of a cell culture assay using fibroblasts for the obtained hydrogel films. Polystyrene dishes (PS dish), commonly used for cell culture, were employed as the control. Hydrogel films containing various concentrations of chlorhexidine were used for the cell culture experiments. During the first 4 h of culture, a slightly higher number of cells were observed on the PS dish (*p* < 0.05). Regarding cell adhesion on the hydrogel samples containing chlorhexidine, no significant changes were observed at this stage. After 24 h, however, a lower number of adherent cells was registered on the PS dish compared to the number of cells found on the hydrogel films (*p* < 0.05). A slight decrease in the number of adhered cells was observed as the chlorhexidine concentration in the hydrogels increased. After 24 h, the difference in the number of attached cells became more evident. At 48 h, a considerable difference was observed between the number of adherent cells on the PS dish and on the hydrogel films, with fewer cells present on the PS dish. For the hydrogels, the decreasing trend in the number of adherent cells with increasing chlorhexidine amount became more pronounced (*p* < 0.05). Finally, after 72 h of culture, a significant difference was found between the number of adherent cells on the PS dish and the hydrogel samples. Although the trend of decreasing cell adhered number with increasing chlorhexidine content was more evident, the number of adherent cells on the hydrogels containing 0.8 wt% chlorhexidine was still substantially higher than that observed on the PS dish. These results suggest that, although chlorhexidine concentration influences the number of adherent cells, the value obtained for the hydrogels remains considerably higher than that of PS dishes (the standard surface for cell adhesion).

The slight suppression of fibroblast adhesion at higher chlorhexidine levels may be related to changes in surface hydrophilicity and protein adsorption behavior. Specifically, an increase in protein adsorption correlated with reduced contact angle (i.e., increased hydrophilicity), while a higher contact angle favored cell attachment, consistent with previous findings [51,52]. Similar studies have reported that fibroblasts adhere more effectively to moderately rigid surfaces with a contact angle near 52°, suggesting that both surface wettability and mechanical stiffness play critical roles in regulating cell–substrate interactions [52]. It has been reported that materials designed for dental applications, such as alginate/gelatin hydrogels, exhibit that material stiffeness and swelling have a negative effect on cell adhesion. Therefore, it is important to futher investigate the effect of chlorhexidine incorporation into the polimeric matrix and its influence on cell adhesion [53,54]. More investigation is required to evaluate the relationship between hydrogel stiffness and cell adhesion. In this context, the observed differences in cell density between hydrogels containing 0.1 and 0.8 wt% chlorhexidine may stem from variations in water adsorption, contact angle, and the reduced stiffness of the hydrogel network caused by the antibacterial agent.

**Figure 7:**
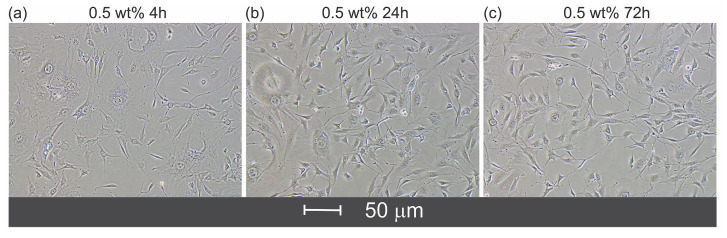
Dependence of fibroblast adhesion on the concentration of chlorhexidine within the hydrogel matrix. Phase-contrast light images of adherent cells on hydrogel films at 4 (**a**), 24 (**b**), and 72 h (**c**) culture time. The images showed higher adherent cells with the increment of cell culture time.

Figure 7 shows the adhesion and proliferation behavior of fibroblasts cultured on hydrogel films containing 0.5 wt% chlorhexidine after different incubation times ranging from 4 to 72 h. A clear dependence of fibroblast attachment on the chlorhexidine concentration within the hydrogel matrix was observed. As presented in Figure 6, increasing the chlorhexidine content suggests a slight reduction in the films’ stiffness, which directly affected cellular migration and adhesion dynamics through mechanotactic responses. Mechanotaxis, the directed migration of cells in response to gradients of substrate stiffness, plays a crucial role in tissue remodeling and wound healing processes. To support this, more investigation into the mechanical and rheological properties of the hydrogels needs to be carried out. Previous studies have reported that fibroblasts preferentially migrate toward stiffer substrates, as these offer greater resistance and provide more favorable mechanical cues for focal adhesion formation and cytoskeletal organization [55,56].

The observed decrease in cell density at higher chlorhexidine concentrations may thus be attributed to a combination of physicochemical and mechanical effects. Chlorhexidine incorporation could alter the internal polymeric network of the hydrogel, reducing crosslinking density and, consequently, lowering the elastic modulus. This decrease could limit the ability of fibroblasts to generate sufficient traction forces for stable adhesion [57]. Additionally, the smoother surface morphology associated with higher chlorhexidine loading may reduce the availability of anchoring sites for extracellular matrix proteins, thereby diminishing integrin-mediated interactions between fibroblasts and the substrate [58]. Together, these effects result in a measurable decline in fibroblast attachment and spreading, suggesting that mechanical properties and surface chemistry are co-regulatory factors in cell–hydrogel interactions.

Although we observed well-extended fibroblasts and an absence of visible morphological signs of cell damage in the hydrogels after 72 h, we acknowledge that microscopy and adhesion metrics alone do not fully replace quantitative viability assays. Previous studies on chlorhexidine-loaded cellulose hydrogels indicate that it can be successfully incorporated and provides sustained antimicrobial activity [48], but this antibiotic is known to exhibit dose-dependent cytotoxicity in vitro [59,60,61,62]. In addition, incorporating chlorhexidine into a polymeric or mineral matrix can mitigate acute toxicity [63]. In context of early onset cytocompatibility, cell density and fibroblast morphology already provide sufficient evidence of cytocompatibility [64]. Our current results are qualitative evidence that the tested formulations promote fibroblast adhesion under the evaluated conditions at early onset cytocompatibility in the first 72 h. Definitive assessment of cytocompatibility will require standard viability/proliferation assays (MTT/CCK-8 and Live/Dead) and their correlation with chlorhexidine release kinetics. This work is included in subsequent studies.

#### 2.3.3. Cell Morphology on Hydrogel Films

**Figure 8:**
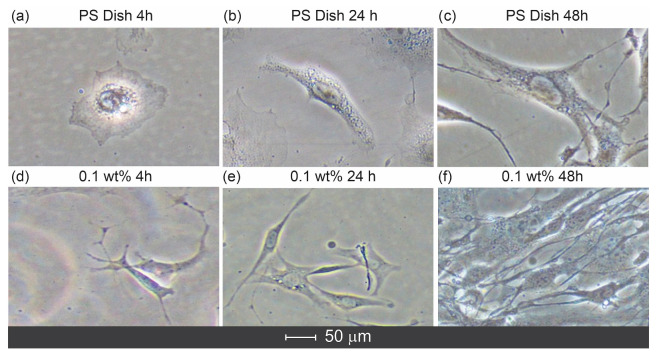
The cellular morphology of fibroblasts cultured on hydrogel films with chlorhexidine evaluated over different culture times. Phase-contrast light images of adherent cells on the hydrogel films. The control group (PS dish) was observed at 4 h (**a**), 24 h (**b**), and 48 h (**c**). Additionally, images of adherent cells on cellulose hydrogel containing 0.1 wt% chlorhexidine were taken at 4 h (**d**), 24 h (**e**), and 48 h (**f**) Adherent cells on PS dish at 4 h of cell culture time showed round-like shape, compared to a more anisotropic shape, observed in adherent cells on cellulose hydrogel.

Figure 8 depicts the cellular morphology of fibroblasts cultured on the hydrogel films compared with those grown on a commercial polystyrene (PS) plate (a–c). Distinct morphological differences were observed between the substrates. In the hydrogel film containing 0.1 wt% chlorhexidine (Figure 8d–f), fibroblasts exhibited firm adhesion and uniform spreading over the surface. After 4 h of culture (Figure 8d), the cells displayed an elongated spindle-like morphology, in contrast to the more rounded shape observed on the PS plate (Figure 8a). The cell boundaries on the cellulose-based hydrogel films appeared well integrated with the substrate, showing diffuse edges indicative of strong adhesion. During the early hours of culture, the fibroblasts on the hydrogel surfaces adopted an anisotropic shape, suggesting active cytoskeletal reorganization guided by the hydrogel’s surface topography and elasticity.

Conversely, fibroblasts cultured on the PS plate maintained a predominantly rounded morphology after 4 h (Figure 8a), implying weak adhesion and limited spreading on the rigid, hydrophobic surface. In comparison, the cells on the hydrogel films (Figure 8e,f) showed greater cytocompatibility, attributed to the soft surface of the film and the cellulose matrix that better mimics the extracellular environment. When the chlorhexidine addition in the hydrogel increased to 0.8 wt%, the films exhibited lower swelling capacity and possibly reduced surface softness, which corresponded with smaller aspect ratios, elongated cell axes, and higher cell density.

The morphological observations indicate that as chlorhexidine content increases in the cellulose-based hydrogel films, key physicochemical changes in the material, such as reduced swelling, increased network density, and altered surface properties, lead to pronounced effects on fibroblast adhesion, shape, and density [65]. More specifically, higher antibacterial agent loading appears to stiffen the hydrogel network by increasing crosslink-like constraints, which decreases swelling and produces a less compliant matrix. Such a stiffer and less swollen environment restricts cellular traction generation and spreading, resulting in lower aspect ratios and more compact shapes [48]. Moreover, surface hydrophilicity and topography are likely to play auxiliary roles. Softer, hydrophilic surfaces provide more favorable conditions for protein adsorption (particularly ECM proteins) and integrin binding, facilitating adhesion and spreading. In contrast, smoother, less swollen surfaces with fewer anchorage points (pores, surface irregularities) reduce the effective surface area and limit focal adhesion formation, hence leading to more rounded fibroblast morphology and reduced cell spreading [66].

**Figure 9:**
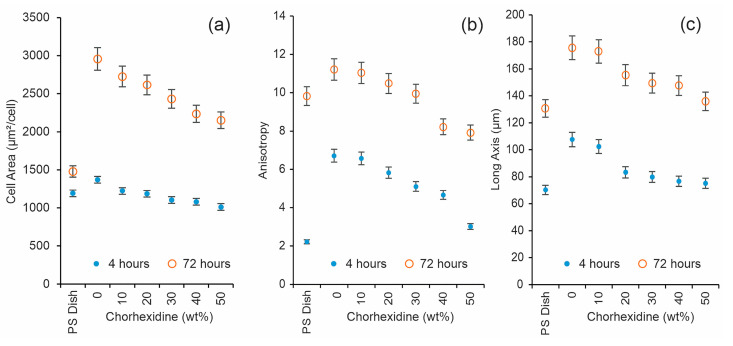
Effects of chlorhexidine concentration on morphological parameters of cell growth on the hydrogel’s surface. (**a**) Cell area, (**b**) anisotropy, and (**c**) long axis, *p* < 0.05, *n* = 6. The measured morphology parameters showed more anisotropic shape and higher cell area at 72 h of cell culture time.

The quantitative analysis of cell morphology revealed that the projected cell area, anisotropy, and major axis length were sensitive to the chlorhexidine content in the hydrogel films, as illustrated in Figure 9. These morphological parameters exhibited a systematic decrease with increasing chlorhexidine concentration, particularly on smoother surfaces. As shown in Figure 9a–c, the projected cell area, anisotropy, and major axis length all diminished progressively as the chlorhexidine content increased. Cell morphology parameters were analyzed at different cell culture times. Figure 7 shows cell morphology after 4 h and 72 h of cell culture, revealing the same tendency with increasing chlorhexidine content. The reduction in projected cell area is consistent with previous studies [36], which reported smaller spreading areas for fibroblasts cultured on smoother or less textured surfaces.

Two mechanistic explanations can account for the observed reduction in cell spreading at higher chlorhexidine loadings. First, chlorhexidine incorporation may alter the surface topography and roughness of the hydrogel, thereby reducing the density of available anchorage sites for cell attachment. Second, this change likely interferes with the formation of focal adhesions, specialized integrin-based complexes that mediate cell binding to adsorbed ECM proteins such as fibronectin and collagen. As the number of functional integrin binding sites decreases on smoother surfaces, fewer focal adhesion complexes can form, weakening cell–substrate interactions and restricting cytoskeletal extension. Consequently, the overall reduction in projected area and cell elongation reflects a limited ability of fibroblasts to establish strong mechanical coupling with the hydrogel surface, highlighting the close interplay between surface topography, biochemical cues, and mechanotransduction processes in determining cell morphology.

Similar correlations between surface smoothness, focal adhesion dynamics, and cell morphology have been reported for various biomaterial systems. For example, fibroblasts cultured on highly polished titanium or polymeric surfaces exhibit smaller adhesion areas and less organized actin stress fibers compared to those on microtextured substrates [67]. In hydrogel-based scaffolds, surface roughness and stiffness synergistically influence integrin clustering and focal adhesion kinase activation, both of which govern cell shape and migration [68]. Therefore, the observed decrease in fibroblast spreading at higher chlorhexidine concentrations supports the notion that surface topography and biochemical composition are critical regulators of cell–material interactions, ultimately dictating cellular morphology, adhesion strength, and tissue integration potential.

It is worth saying that the combined assessment of fibroblast adhesion, cell morphology, and protein adsorption provides valuable preliminary information on the early onset cytocompatibility of cellulose–chlorhexidine hydrogels. Similar studies have used adhesion and extension parameters in polysaccharide hydrogels to infer favorable cell–material interactions, especially when comprehensive viability studies have not yet been conducted [69]. Furthermore, chlorhexidine is known to exhibit dose-dependent cytotoxicity in vitro; for example, fibroblast survival decreases drastically at concentrations ≥ 0.02% after brief exposure [65]. In our study, the observation of adherent and well-extended fibroblasts after 72 h, along with the absence of evident cytotoxic morphological markers (such as cell rounding or detachment), suggests that the tested hydrogel formulations promote cell adhesion under the evaluated conditions. However, quantitative viability and proliferation assays are still needed to fully validate biocompatibility. Future research will focus on correlating chlorhexidine release kinetics, the hydrogel’s mechanical/structural parameters, and detailed viability and proliferation measurements for relevant applications in dentistry.

## 3. Conclusions

Cellulose-based hydrogels were successfully developed from pineapple waste using the LiCl/DMAc solvent system, demonstrating an environmentally sustainable pathway for the production of biomedical materials. The incorporation of chlorhexidine conferred antibacterial properties, slightly modifying surface morphology, swelling capacity, and mechanical performance. These structural adjustments subtly influenced fibroblast adhesion and protein adsorption without compromising overall early onset cytocompatibility. The chlorhexidine-loaded cellulose hydrogels combined effective antimicrobial action with adequate mechanical stability and hydration behavior, key characteristics for potential dental and soft tissue applications. Chlorhexidine acted not only as an antiseptic but also as a structural modulator within the hydrogel matrix, influencing cell–material interactions. By controlling the chlorhexidine concentration, the system can be adjusted to achieve a balance between antibacterial efficacy and cell compatibility. Overall, these findings highlight a sustainable and functional biomaterials platform derived from agro-industrial waste, which offers promising potential for the future development of materials for infection control and tissue support in regenerative dentistry.

## 4. Materials and Methods

### 4.1. Materials

Pineapple waste fibers were obtained from a food manufacturing company in Chihuahua State, Mexico. N,N-dimethylacetamide (DMAc), lithium chloride, ethanol, sodium hydroxide, potassium hydroxide, sodium hypochlorite, sulfuric acid, and chlorhexidine were purchased from Sigma-Aldrich (St. Luis, MO, USA). Bicinchoninic acid (BCA) kit was delivered by Sigma-Aldrich (St. Luis, MO, USA ). Fetal bovine serum (FBS) and bovine serum albumin (BSA), purchased from Sigma Aldrich, phosphate-buffered saline (PBS, Dullbecco Co., Ltd., Montréal, QC, Canada), 0.05 *w*/*v*% trypsin-0.053 M-ethylenediaminetetraacetate (trypsin-EDTA), were supplied by Gibco (Tokyo, Japan), and formaldehyde (37 vol% aqueous solution) and phosphate-buffered saline (PBS) were purchased from Wako Co., Ltd. (Tokyo, Japan). NIH3T3 mouse embryonic fibroblast cells were acquired from Vitrogen (Tokyo, Japan).

### 4.2. Hydrogel Preparation

Pineapple waste, including peels, leaves, and cores, represents a promising raw material because of its high availability and high cellulose content. Pineapple fiber waste was treated as follows: firstly, the waste was thoroughly washed multiple times with distilled water to eliminate any residual sugars from the manufacturing process. The fiber was then dried in an oven at 60 °C. Afterward, 3 g of the fiber were immersed in 1000 mL of 10 vol% NaOH solution and stirred at 100 °C for 12 h, resulting in a black liquor solution [36]. After this process, the fiber was washed extensively with distilled water until the pH reached 7. The fiber was then recovered through filtration and transferred to a container with 1000 mL of 4% H_2_SO_4_ solution, while being magnetically stirred at 100 °C for 2 h. The fiber underwent another washing with distilled water until it returned to a neutral pH. Subsequently, the treated fiber was soaked in 1000 mL of 10 vol% NaOCl solution, a bleaching agent, for 2 h to achieve a lighter color [35]. After fiber treatment, around 60% of the bulk mass of bagasse pineapple was obtained as cellulose fibers. Finally, the fiber was washed again with distilled water and then dried under vacuum for two days.

To obtain a cellulose solution from the treated pineapple fiber, 1 g of the fiber was suspended in 300 mL of distilled water and stirred overnight to ensure thorough swelling. The fiber was then recovered by filtration using filter paper. After that, it was placed in 300 mL of ethanol and stirred magnetically for 24 h. Once again, the fiber was recovered by filtration and dried in an oven at 60 °C for 24 h. For the solution, the treated fiber was added to a 6 wt% LiCl in DMAc solvent system [36], stirring magnetically at room temperature for three days until a viscous solution was formed.

Samples of cellulose with chlorhexidine were prepared in concentrations ranging from 0.1 to 0.8 wt%. This range was selected based on various dental pharmaceutical formulations [30,32]. Transparent solutions were successfully achieved. Hydrogel films containing chlorhexidine were prepared by pouring 10 g of the cellulose solution into a glass tray (10 cm in diameter) and allowing it to sit for 12 h in a container with 20 mL of ethanol for coagulation. This process, known as the phase inversion method, resulted in transparent hydrogel films. The films were then washed several times with ethanol and subjected to a shaking bath for 36 h to remove DMAc. The hydrogel films were subsequently immersed in distilled water overnight and stored in PBS at 4 °C in a plastic container.

### 4.3. Hydrogel Characterization

The mechanical properties, tensile strength (*σ*) and elongation at break (*ε*), were characterized using a universal testing machine (LTS-500N-520, Minebea Mitsumi, Tokyo, Japan) equipped with a 2.5 kN load cell. Rectangular hydrogel films (1 mm × 10 mm × 50 mm) were carefully cut and subjected to uniaxial tension along their longitudinal axis under ambient conditions. For the mechanical characterization of the hydrogels, five specimens were evaluated and averaged to ensure reproducibility. Three tests were also performed for each chlorhexidine concentration to confirm experimental consistency. σ and ε were calculated according to Equations (1) and (2).

(1) $$\sigma (\frac{N}{{\mathrm{mm}}^{2}})=\frac{F}{A}$$

(2) $$\varepsilon \left(\%\right)=\frac{∆L}{L_{i}}\;\times \;100$$

*F* and *A* stand for the maximum load (N) and cross-sectional area (mm^2^), respectively, and Δ*L* is the length difference (Final length-Initial length), and *L_i_* is the initial length.

The equilibrium water content (*EWC*) of the films was evaluated to understand their water adsorption properties. *EWC* of a hydrogel is defined as the fraction of water that the hydrogel contains at full hydration. To assess this, specimens were prepared as follows: dehydrated hydrogel samples measuring 5 mm × 5 mm were cut and immersed in distilled water for 36 h to allow for rehydration. Subsequently, the samples were removed from the container, and excess water was removed with blotting paper before recording the weight of the rehydrated films. Equation (3) was used to determine the *EWC*.

(3) $$EWC\left(100\right)=\left[\left(\frac{W_{s}-W_{d}}{W_{s}}\right)\right]\times 100$$

*W_s_* and *W_d_* are, respectively, the weight of the swollen samples and the dry weight of the sample.

The surface wettability of the hydrogels was determined by measuring the static water contact angle using a contact angle analyzer (DMo-502WA, Kyowa Interface Science Co., Ltd., Saitama, Japan). Square hydrogel specimens (20 mm × 20 mm) were placed on a clean and leveled glass stage prior to the measurement. A 3 μL droplet of distilled water was deposited onto the sample surface using an automated microsyringe. The contact angle between the droplet and the hydrogel surface was recorded immediately with the instrument’s software. For each formulation, three independent samples were tested, and at least three measurements were performed per sample. The average value and standard deviation were calculated to report the final contact angle.

Hydrogels with chlorhexidine were analyzed by UV–Vis spectroscopy (V-520, Jasco, Tokyo, Japan) in the range of 190 to 400 nm. For the hydrogel films, containing different chlorhexidine contents, samples were cut into slices and sandwiched between quartz plates to run the spectra.

The microstructural features of the hydrogels were examined using a scanning electron microscope (SU3500, Hitachi, Tokyo, Japan) operated at an accelerating voltage of 15 kV. Before the analysis, the samples were freeze-dried (BK-FD10PT, BIOBASE, Shandong, China) at −60 °C under vacuum for 24 h to ensure complete removal of residual moisture. The dried hydrogels were subsequently immersed in liquid nitrogen and immediately fractured by applying a quick impact on a sharp edge using a metal tool to expose the internal cross-section. The fractured specimens were mounted on aluminum stubs and coated with a thin layer of gold using a sputter coater (E1010, Hitachi, Tokyo, Japan) to enhance surface conductivity. SEM micrographs were captured at various magnifications to evaluate the cross-sectional morphology of the hydrogels.

### 4.4. Antimicrobial Assay

For the antimicrobial evaluation of the hydrogels, *Streptococcus mutans* (*S. mutans*), *Streptococcus sanguis* (*S. sanguis*), and *Streptococcus anginosus* (*S. anginosus*) were used as representative oral bacterial strains [25,30]. Bacterial suspensions were prepared and adjusted to an optical density of 0.1 at 600 nm using a spectrophotometer (Model 7305, Jenway, Vernon Hills, IL, USA), corresponding to approximately 1 × 10^8^ CFU/mL.

Müller–Hinton agar was prepared, sterilized at 120 °C, and poured into sterile Petri dishes. Circular hydrogel discs (5 mm in diameter) were cut from each formulation and sterilized under UV light for 25 min in advance of the testing. Hydrogel films without chlorhexidine were used as controls. For each bacterial strain, 100 µL of the standardized inoculum was evenly spread onto the agar surface using sterile cotton swabs. The hydrogel discs were then placed on the inoculated agar and incubated at 37 °C for 24 h. Antimicrobial activity was assessed by measuring the diameter of inhibition zones around each disc after 15, 24, and 48 h of incubation.

A colony-forming unit (CFU) count was performed using tubes containing 5 mL of sterilized Muller–Hinton broth. Each tube was inoculated with *Streptococcus mutans* (*S. mutans*), *Streptococcus sanguis* (*S. sanguis*), and *Streptococcus anginosus* (*S. anginosus*) at an optical density of 0.1 absorbance units, measured with a UV–Vis spectrophotometer at 600 nm. Hydrogel samples (1 cm × 1 cm) were cut and sterilized under UV light for 25 min. Samples containing various amounts of chlorhexidine were then placed in the tubes containing Muller–Hinton broth and incubated at 37 °C for 24 h. Tubes containing only Muller–Hinton broth and a tube containing the hydrogel film without chlorhexidine were used as controls. After 24 h, serial dilutions (1:1,000,000) were prepared. From each dilution, 100 µL were placed onto Muller–Hinton agar and streaked using a Drigalski loop. The agar plates were incubated at 37 °C for 24 h, after which the colony count was performed.

### 4.5. Protein Adsorption

Protein adsorption on the hydrogel surfaces was quantified using the bicinchoninic acid (BCA) assay following a previously established protocol [35]. Square hydrogel specimens (5 mm × 5 mm) were immersed in 1 mL of phosphate-buffered saline (PBS) containing 1 mg/mL bovine serum albumin (BSA) and incubated at 37 °C for 4 h. To evaluate protein adsorption under more physiologically relevant conditions, additional samples were incubated in 1 mL of Dulbecco’s Modified Eagle Medium (DMEM) supplemented with 10 vol% fetal bovine serum (FBS) under the same conditions.

After incubation, the hydrogels were rinsed three times in PBS for 10 min each to remove unbound proteins. Subsequently, 2 mL of a 2 wt% sodium dodecyl sulfate (SDS) solution was added to each sample, and the mixtures were gently agitated in a shaking bath at 25 °C to desorb the adsorbed proteins. The protein concentration in the resulting solutions was determined spectrophotometrically at 562 nm using a calibration curve prepared from standard BSA solutions [36]. All measurements were performed in triplicate, and data from six independent experiments were averaged for analysis.

### 4.6. Cell Culture

For the biocompatibility assessment, mouse embryonic fibroblasts (NIH3T3 cell line) were employed following a previously established procedure [36]. Circular hydrogel films (30 mm in diameter) were prepared and sterilized by immersion in 70 vol% ethanol for 30 min, followed by rinsing with sterile phosphate-buffered saline (PBS) to eliminate residual ethanol.

A fibroblast suspension was prepared in Dulbecco’s Modified Eagle Medium (DMEM) supplemented with 10% fetal bovine serum and antibiotics, adjusting the cell density to 8 × 10^3^ cells/cm^2^. Each hydrogel sample was seeded with 2 mL of this suspension and incubated at 37 °C in a humidified atmosphere containing 5% CO_2_. Cell adhesion and morphology were evaluated after 4, 24, 48, and 72 h of incubation using optical microscopy. At each time point, the number of adherent cells and their morphological features, such as spreading and elongation, were quantified. Four replicates were analyzed for each hydrogel formulation, including control samples maintained under identical culture conditions.

### 4.7. Cell Morphology

For the cytotoxicity assessment, the morphology of fibroblasts adhered to the hydrogel surfaces was examined using an inverted optical microscope (CKX4, Olympus, Tokyo, Japan). Hydrogel samples were collected after 4, 24, 48, and 72 h of cell culture, following previously described procedures [36]. After each incubation period, the Petri dishes containing the hydrogel samples were gently rinsed twice with 2 mL of phosphate-buffered saline (PBS) to remove non-adherent cells. Subsequently, 2 mL of 3.7 vol% formaldehyde solution was added to fix the remaining adherent cells onto the hydrogel surface.

Each dish was divided into four quadrants, and twenty micrographs were captured per quadrant using the CellSens imaging software (Olympus) version 1.12. Morphological parameters, including aspect ratio, projected cell area, and major axis length, were quantified from the acquired images. All analyses were performed in six independent experiments to ensure statistical reliability.

### 4.8. Statistical Analysis

Statistical analyses were carried out using the nonparametric Kruskal–Wallis test, followed by Dunn’s post hoc comparison, to assess the influence of chlorhexidine concentration on the physicochemical and biological properties of the hydrogels. Differences were considered statistically significant at *p* < 0.05. All data processing was performed using IBM SPSS Statistics software, version 25 (IBM Corp., Armonk, NY, USA). Each experiment was conducted in triplicate, and results were expressed as the mean ± standard deviation (SD). This statistical approach was chosen because the data did not follow a normal distribution, allowing for reliable comparison of mechanical strength, biocompatibility, cytotoxicity, and antimicrobial performance among the hydrogel formulations.

## Figures and Tables

**Table 1:** Effect of chlorhexidine loading on the properties of hydrogel films. Samples were tested at 25 °C. Data are presented in mean and ± standard deviation.

Chlorhexidine (wt%)	Water Content (%)	Contact Angle	Tensile Strength (N/mm^2^)	Elongation (mm)	Shear Viscosity (Cp)	
					6 rpm	60 rpm
0.0	34 ± 0.5	51 ± 0.3	0.67 ± 0.4	38 ± 0.5	412 ± 1	407 ± 0
0.1	31 ± 0.3	53 ± 0.2	0.65 ± 0.6	36 ± 0.3	409 ± 2	405 ± 3
0.2	27 ± 0.5	55 ± 0.8	0.62 ± 0.4	33 ± 0.1	387 ± 4	382 ± 1
0.4	25 ± 0.2	56 ± 0.3	0.59 ± 0.2	30 ± 0.4	351 ± 1	348 ± 2
0.5	24 ± 0.6	60 ± 0.6	0.57 ± 0.3	26 ± 0.2	275 ± 4	273 ± 3
0.8	22 ± 0.2	63 ± 0.1	0.54 ± 0.2	20 ± 0.3	206 ± 1	201 ± 2

## Data Availability

All data obtained during this study can be found in the research archives of the Master’s Program in Dental Sciences of the Autonomous University of Ciudad Juarez and can be requested from the corresponding author.
